# Case report: Primary pleural low-grade fibromyxoid sarcoma in a 4-year-old boy with molecular confirmation

**DOI:** 10.3389/fonc.2023.1269078

**Published:** 2023-12-20

**Authors:** Xiangni He, Wenyi Jing, Xin He, Min Chen, Hongying Zhang

**Affiliations:** Department of Pathology, West China Hospital, Sichuan University, Chengdu, China

**Keywords:** low-grade fibromyxoid sarcoma, pleural tumor, pediatric sarcoma, *FUS::CREB3L2* gene fusion, molecular analysis

## Abstract

Low-grade fibromyxoid sarcoma (LGFMS) is a rare malignant fibroblastic tumor, principally affecting the deep tissues of the proximal trunk and extremities in young adults. However, primary pleural LGFMS is extremely rare, and only three cases have been reported in the previous English literature without genetic confirmation. Furthermore, the historical pleural LGFMS cases were all adults, and the primary pleural LGFMS in children has never been reported to date. Here, we presented a primary pleural LGFMS in a 4-year-old boy with detailed clinical, pathological, and molecular results. Histologically, the current tumor showed typical alternating collagenous and myxoid areas, containing spindled or oval tumor cells arranged in a whorled and short fascicular pattern. In some areas, the tumor cells exhibited moderate atypia, and mitotic figures were identified but without the identification of giant collagen rosettes. Immunohistochemically, all the neoplastic cells showed strong and diffuse positivity for MUC4. Genetically, *FUS* gene rearrangement was revealed by fluorescence *in-situ* hybridization (FISH), and subsequently, next-generation sequencing (NGS) and polymerase chain reaction (PCR) further demonstrated the *FUS::CREB3L2* fusion transcript. To the best of our knowledge, this is the first case of primary pleural LGFMS with the identification of *FUS* gene rearrangement and *FUS::CREB3L2* fusion in a 4-year-old child. Our study expands the age range of pleural LGFMS and highlights the combination of morphological, immunohistochemical, and molecular analyses in such challenging cases.

## Introduction

Low-grade fibromyxoid sarcoma (LGFMS) is a rare malignant fibroblastic neoplasm, mainly occurring in the deep tissues of the proximal trunk and extremities. The tumors typically arise in young adults with a slight male predilection, and approximately 20% of cases are <18 years of age (1–3). Morphologically, classic LGFMS is composed of fibrous and myxoid areas, with bland, spindled cells in a whorled or fascicular growth pattern, and easy to be misdiagnosed as a benign tumor by histology only. Immunohistochemically, MUC4 has been reported as a sensitive and specific marker for the diagnosis of LGFMS. Genetically, more than 90% of LGFMS cases harbor t(7;16)(q33; p11) translocation, resulting in *FUS::CREB3L2* fusion gene, and a minority of the cases contain *FUS::CREB3L1* or *EWSR1::CREB3L1* fusions. The identification of *FUS::CREB3L2* or other rare fusions by molecular analysis could aid in the diagnosis of LGFMS (4–6).

In recent years, it has been described that LGFMS can also arise from other unusual sites, including the head/neck, abdominopelvic, retroperitoneal, gastrointestinal viscera, and mediastinum. It is worth highlighting that primary pleural LGFMS is extremely rare. To the best of our knowledge, only a total of three cases of primary pleural low-grade fibromyxoid sarcoma have been reported to date, and all lacked genetic validation (7–9).

We presented a primary pleural LGFMS case with the identification of *FUS* gene rearrangement by fluorescence *in-situ* hybridization (FISH) and the *FUS::CREB3L2* fusion gene by next-generation sequencing (NGS) and polymerase chain reaction (PCR). Moreover, although 20% of LGFMS cases aged <18 years, primary pleural LGFMS in children has never been reported before. Here, we reported the first genetically confirmed pleural LGFMS case in a 4-year-old boy, with detailed clinical, pathological, and molecular information.

## Case presentation

**Figure 1:**
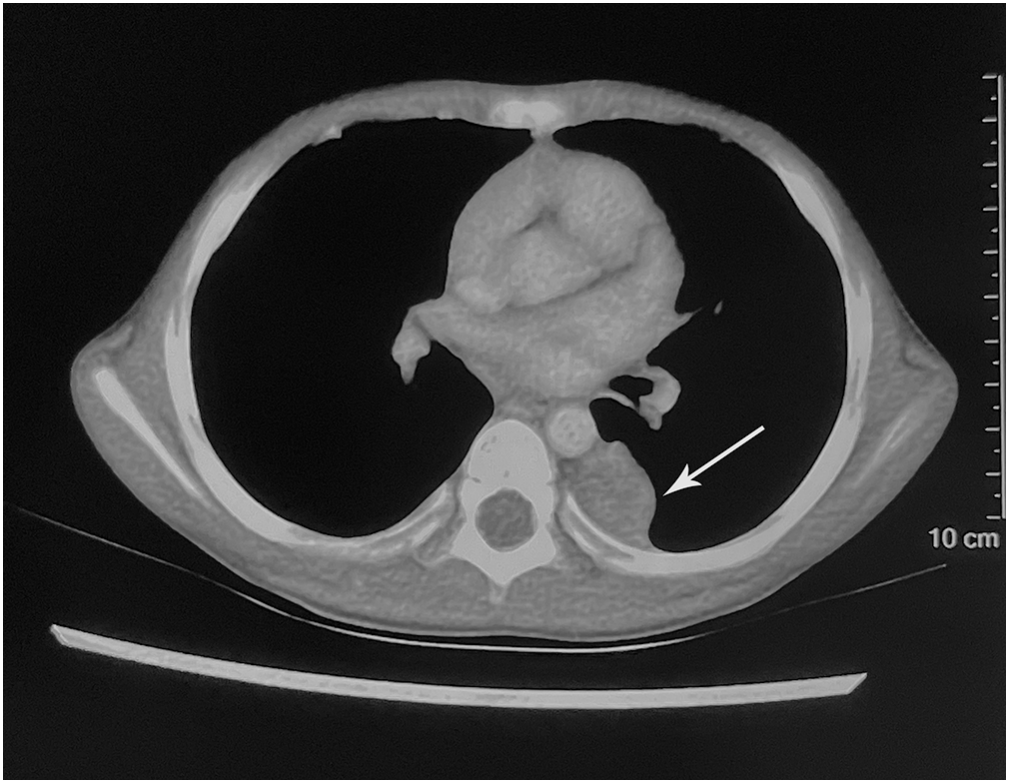
Imaging findings. The contrast-enhanced computed tomography showed a hypodense mass occupying the left side of the posterior mediastinal paravertebral region (arrow).

A 4-year-old boy was admitted to a local hospital with a 1-month history of cough and fever and diagnosed with “pneumonia.” Contrast-enhanced computed tomography (CT) revealed a well-defined, heterogeneous soft tissue mass measuring 2.2 cm × 1.9 cm, occupying the left side of the posterior mediastinal paravertebral region (**Figure 1**).

The patient was admitted to the department of pediatric surgery, and surgery was performed in August 2022. During the operation, a well-circumscribed mass was identified under the visceral pleura and clearly demarcated from the lung parenchyma. The patient underwent complete resection of the mass. Grossly, this lesion revealed a well-demarcated mass measuring 5 cm at its greatest dimension. On the cut surface, the mass demonstrated a white color and a brittle texture without obvious necrosis. The pathologists of the peripheral hospital first considered the diagnosis of the current case as a spindle-cell proliferative lesion, which was inclined to the tumor, and they suggested consultation to further clarify the diagnosis.

**Figure 2:**
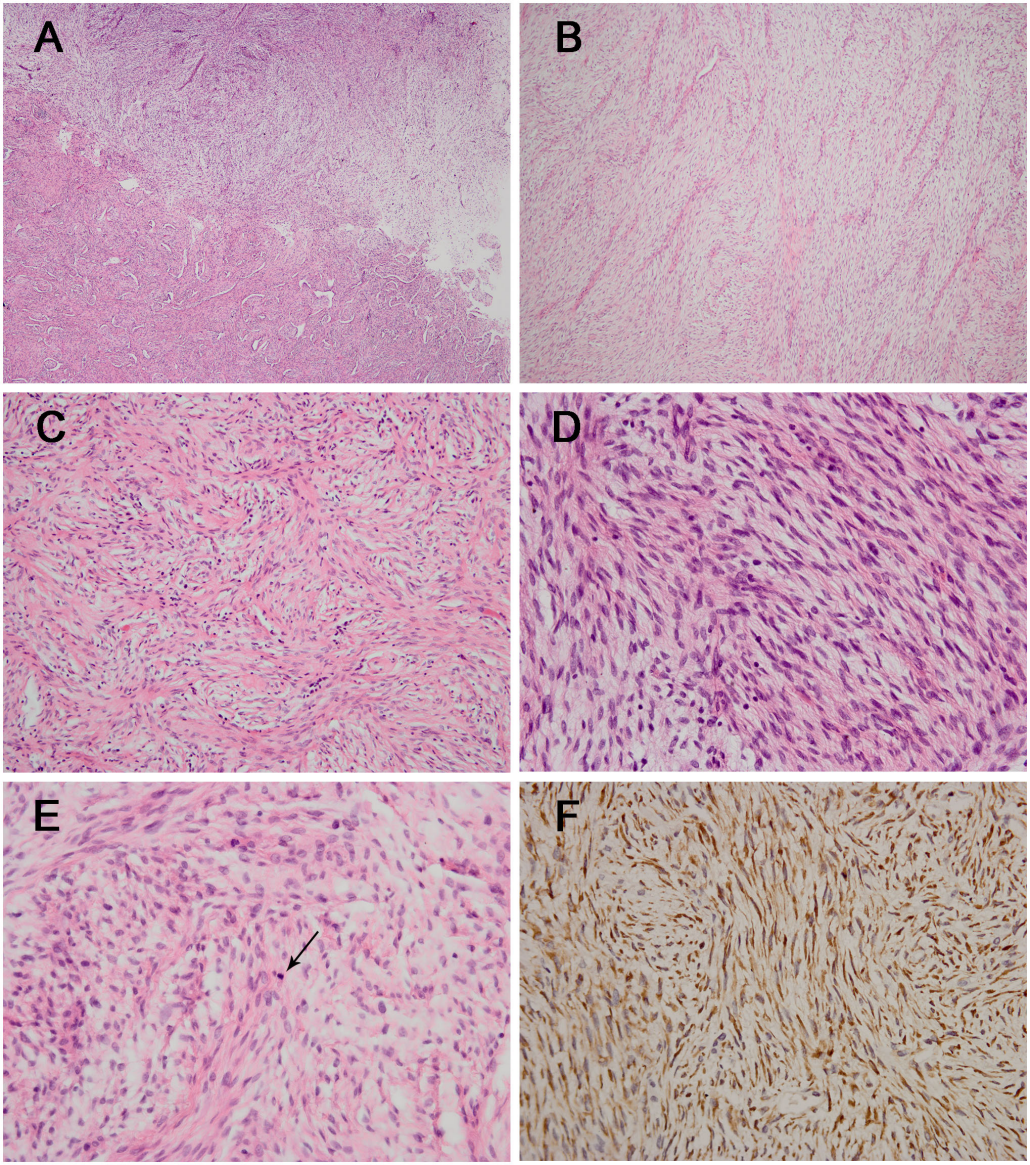
The histological and immunohistochemical results of the tumor. **(A)** The lesion was composed of fibrous stroma and myxoid areas with an abrupt transition [hematoxylin and eosin staining (H&E); magnification: ×40]. **(B)** The classic myxoid areas and fibrous stroma were shown in the tumor (H&E; magnification: ×100). **(C)** Neoplastic cells were spindle-shaped and arranged in a whorled and short fascicular pattern (H&E; magnification: ×200). **(D)** Some areas had increased cellularity with moderate atypia (H&E; magnification: ×400) with the identification of mitotic figure (arrow) [**(E)** H&E; magnification: ×400]. **(F)** The tumor cells showing diffuse and strong positivity for MUC4 (magnification: ×400).

Our department received consultation slides from the peripheral hospital. Microscopically, the lesion was relatively well delineated and composed of fibrous stroma and myxoid areas with an abrupt transition (**Figures 2A, B**). The tumor cells were spindle and oval-shaped, arranged in a whorled and short fascicular pattern (**Figure 2C**). In some areas, the lesion had increased cellularity, and the neoplastic cells had spindled hyperchromatic nuclei showing moderate atypia, with indistinct pale eosinophilic cytoplasm and a few visible nucleoli (**Figure 2D**), and mitotic figures were identified without atypical forms (2/10 high power fields) (**Figure 2E**). Immunohistochemically, all the tumor components stained diffusely and strongly positive for MUC4 (**Figure 2F**). The neoplastic cells were negative for desmin, S-100, STAT-6, myogenin, CD34, ERG, TLE1, β-catenin, EMA, myoD1, SOX10, ALK, TRK, SMA, and loss of H3K27me3. The Ki-67 (MIB-1) index was 5% of these cells.

**Figure 3:**
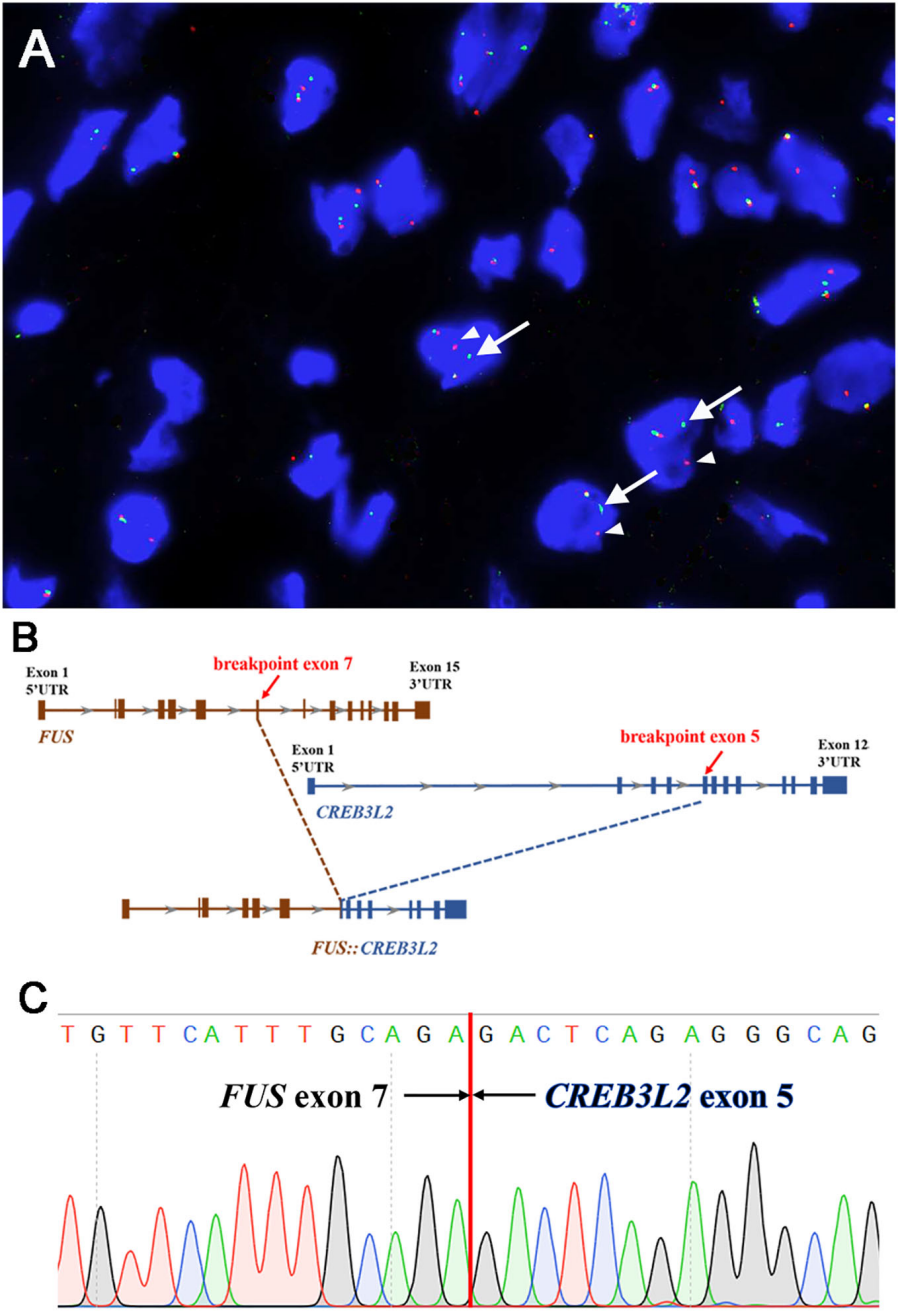
Molecular findings. **(A)** Fluorescence *in-situ* hybridization demonstrated the presence of *FUS* gene rearrangement in the neoplastic cells [separation of the red (white arrowhead) and green (white arrow) signals]. **(B)** Next-generation sequencing-based technology revealed the breakpoint of *FUS* (exon 7) and *CREB3L2* (exon 5) fusion transcript. **(C)** Sanger sequencing analysis confirmed the presence of the *FUS::CREB3L2* fusion gene.

FISH analyses for *FUS*, *EWSR1*, and *MDM2* were performed using the GSP *FUS* gene probe (Anbiping, Guangzhou, China), GSP *EWSR1* gene probe (Anbiping, Guangzhou, China), and GSP *MDM2* (12q15) Gene Amplification probe (Anbiping, Guangzhou, China). The FISH results demonstrated the presence of *FUS* gene rearrangement (**Figure 3A**). In addition, the tumor was negative for *EWSR1* gene rearrangement and *MDM2* gene amplification.

Subsequently, next-generation sequencing (NGS)-based technology (1,084 cancer-relevant genes, hybrid DNA/RNA panels, Genetron Health, Beijing, China) was applied to the formalin-fixed paraffin-embedded tissue of the lesion. Notably, a *FUS::CREB3L2* fusion was identified at both the DNA and RNA levels. A fusion transcript of *FUS* (exon 7, transcript NM_004960.3) and *CREB3L2* (exon 5, NM_194071.4) was discovered (**Figure 3B**). Then, we further validated the presence of the *FUS::CREB3L2* fusion transcript by PCR (polymerase chain reaction) and Sanger sequencing using primers (*FUS*-forward: 5′-GTCTGATTGTTCATTTGCAGA-3′ and *CREB3L2*-reverse: 5′-GACTCAGAGGGCAGCCTGAGT-3′) (**Figure 3C**). Based on the histological, immunohistochemical, and molecular results, the pleural tumor was diagnosed as LGFMS.

Considering that LGFMS mainly occurs in the deep tissues of the proximal trunk and extremities, a complete physical examination was performed again in this case to exclude the possibility that the pleural LGFMS was a metastasis from the trunk or extremities and revealed no palpable mass. PET-CT was carried out to further rule out the possibility that the pleural tumor was a metastasis and revealed no other lesions. Finally, combining the clinical, radiological, and pathological findings and molecular results, the diagnosis was primary LGFMS of the pleura. At the most recent follow-up, 11 months following the surgery, the patient was in a good status with no evidence of disease.

This study was approved by the West China Hospital Institutional Review Board.

## Discussion

LGFMS mainly occurs in young adults, and approximately 20% of cases are <18 years of age. However, LGFMS arising in children aged <5 years is extremely rare. A SNOMED search of the West China Hospital surgical pathology files from July 2008 to January 2023 has identified 36 LGFMS cases, while only 2 cases younger than 5 years old were detected. Subsequently, we carefully reviewed the previously reported LGFMSs in English literature, and 122 cases (before 18 years old) have been described. Moreover, only 22 cases (22/122, 18.0%) under 5 years old were reported (10–21). Among the 22 cases, the tumors mainly occurred in the extremities (8/22, 36.5%), followed by the trunk (7/22, 31.8%), head and neck regions (5/22, 22.7%), liver (1/22, 4.5%), and abdominal cavity (1/22, 4.5%). To the best of our knowledge, the current case is the first primary LGFMS case that occurred in the pleura of this age group (under 18 years old) in English literature.

In fact, primary pleural LGFMS is exceedingly scarce and only three cases have been reported before (7–9). In 2005, Kim et al. reported the first pleural LGFMS in a 37-year-old man, and the second case was described in a 42-year-old woman. However, MUC4 immunostaining and molecular testing were not performed in these cases. Recently, Perez et al. reported pleural LGFMS in a 32-year-old man, with MUC4 positivity. Our case is the first pleural LGFMS in a 4-year-old child that was immunohistochemically and genetically confirmed.

Furthermore, primary intrathoracic LGFMS is also very rare, and only 27 cases have been reported before (including the current case) (**Table 1**) (7–9, 22–42). The historical intrathoracic cases were composed of 13 male and 14 female patients, aged 4 to 77 years old (median 35 years). These tumors involved the mediastinum (10/27, 37.0%), lung parenchyma (10/27, 37.0%), pleura (4/27, 14.8%), heart (1/27, 3.7%), intrathoracic/pericardium (1/27, 3.7%), and right heart/epicardium (1/27, 3.7%). It needs to be pointed out that intrathoracic LGFMS could sometimes be extremely large, and it may be difficult to determine where the lesion arises from. These results indicated that primary intrathoracic LGFMS cases, especially the pleural lesions, are exceedingly rare, and the diagnoses of LGFMS in such sites are extremely challenging.

**Table 1:** Clinicopathologic and molecular features of previously reported primary intrathoracic LGFMS cases.

Case no.	References	Age	Sex	Symptoms	Location	Size (cm)	Histological features	IHC		Genetic results				Treatment	Outcome
								MUC4	Ki-67	FISH			Sequencing		
										*FUS*	*ESWR1*	Others			
1	Current case	4	M	Cough	Pleura	5	Classic LGFMS	(+)	5%	(+)	(−)	*MDM2* amplification (−)	*FUS*::*CREB3L2* (NGS and PCR)	Complete resection	11 mo/NED
2	Perez, D. et al. (7)	32	M	Cough	Pleura	11	LGFMS with collagen rosettes	(+)	ND	ND	ND	*SYT* rearrangement (−)	ND	Surgical resection	29 mo/NED
3	Liang, W. et al. (8)	42	F	Cough, shortness of breath	Pleura	11	Classic LGFMS	ND	ND	ND	ND	ND	ND	Marginal excision, radiotherapy	NA
4	Kim, S. Y. et al. (9)	37	M	Chest pain, dyspnea	Pleura	NA	Classic LGFMS	ND	ND	ND	ND	ND	ND	Surgical resection	NA
5	Ershadi, R. et al. (22)	26	F	Chest pain, shortness of breath	Left lung	30	LGFMS with collagen rosettes and epithelioid cell	ND	Low	ND	ND	ND	ND	Surgical resection	6 mo/NED
6	Yoshimura, R. et al. (23)	22	M	Asymptomatic	Right upper lung	4.5	LGFMS with collagen rosettes	(+)	ND	(−)	ND	ND	ND	Surgical resection	NA
7	Bartuma, H. et al. (24)	77	M	NA	Right upper lung	5	LGFMS with collagen rosettes	ND	ND	(+)	ND	ND	*FUS*::*CREB3L2* (PCR)	Surgical resection	NA
8	Sargar, K. et al. (25)	12	M	NA	Left lower lung	NA	NA	ND	ND	ND	ND	ND	ND	NA	NA
9	Oramas, D. M. et al. (26)	37	F	Cough, dyspnea, chest pain	Left lower lung	2.4	LGFMS with collagen rosettes	ND	ND	ND	ND	ND	ND	Surgical resection	6 mo/NED
10	Oramas, D. M. et al. (26)	42	M	Cough, dyspnea, chest pain	Right upper lung	3	LGFMS with collagen rosettes	ND	ND	ND	ND	ND	ND	Surgical resection	14 mo/NED
11	Magro, G. et al. (27)	20	F	Asymptomatic	Lung	2	LGFMS with collagen rosettes	ND	ND	ND	ND	ND	ND	Biopsy	12 mo/AWD
12	Kim, L. et al. (28)	50	F	Chest pain	Left lower lung	7.5	LGFMS with collagen rosettes	ND	ND	ND	ND	ND	*FUS*::*CREB3L2* (PCR)	Surgical resection	NA
13	Whale, K. et al. (29)	66	F	Asymptomatic	Right lung	2.6	LGFMS with collagen rosettes and SEF area	ND	2%	(+)	ND	ND	ND	Surgical resection	NA
14	Kurul, İ. C. et al. (30)	16	F	Pain in the arms and legs	Lung	10	Classic LGFMS	ND	ND	ND	ND	ND	ND	Surgical resection	24 mo/NED
15	Mustafa, S. et al. (31)	44	F	NA	Mediastinum	12	LGFMS with collagen rosettes	ND	ND	(+)	ND	ND	ND	Surgical resection	NED
16	Xie, Y. et al. (32)	32	F	Shortness of breath, cough	Mediastinum	14	Classic LGFMS	ND	ND	ND	ND	ND	ND	Surgical resection	36 mo/NED
17	Sajid, M. I. et al. (33)	26	M	Fever, vomiting	mediastinum	17	LGFMS with collagen rosettes	(+)	ND	ND	ND	ND	ND	Surgical resection	22 mo/NED
18	Maeda, E. et al. (34)	50	M	Asymptomatic	Superior mediastinum	13	Classic LGFMS	ND	ND	ND	ND	ND	ND	Surgical resection	60 mo/NED
19	Maeda, E. et al. (34)	19	F	Asymptomatic	Anterior mediastinum	23.5	LGFMS with collagen rosettes	ND	ND	ND	ND	ND	ND	Surgical resection	Recurrence at 60 mo, resected; 60 mo/AWD
20	Takanami, I. et al. (35)	35	M	Asymptomatic	Anterior mediastinum	9	Classic LGFMS	ND	ND	ND	ND	ND	ND	Surgical resection	Recurrence at 108 mo, resected; 110 mo/NED
21	Aissaoui, R. et al. (36)	29	M	Dyspnea	Mediastinum	18.9	Classic LGFMS	ND	ND	ND	ND	*FUS::DDIT3* (−) *ESWR*1::*DDIT3* (−)	ND	Biopsy	NA
22	Williams, C. M. et al. (37)	50	M	Shortness of breath	Mediastinum	NA	LGFMS with epithelioid, round cell	ND	50%	ND	ND	ND	*FUS*::*CREB3L2* (NGS)	Biopsy, chemotherapy	5 mo/died of pericardial effusion and tamponade
23	Galetta, D. et al. (38)	41	M	Asymptomatic	Anterior mediastinum	8	LGFMS with collagen rosettes	ND	ND	ND	ND	ND	ND	Surgical resection radiotherapy	35 mo/NED
24	Gülhan, S. Ş. E. et al. (39)	25	F	Back pain and dyspnea	Posterior mediastinum	17	LGFMS with collagen rosettes	ND	ND	ND	ND	ND	ND	Surgical resection	NA
25	Chan, Y. C. et al. (40)	9	F	Cough and weight loss	Intrathoracic/pericardium	27	Classic LGFMS	(+)	ND	(+)	ND	ND	*FUS*::*CREB3L2* (NGS)	Chemotherapy, surgical resection	12 mo/NED
26	Jakowski, J. D. et al. (41)	44	F	Cough, retrosternal discomfort	Right heart/epicardium	12	LGFMS with collagen rosettes	ND	ND	(+)	ND	ND	ND	Surgical resection	7 mo/NED
27	Ferlosio, A. et al. (42)	57	F	Right cardiac failure	Right ventricle	5	Classic LGFMS (primary); LGFMS with pleomorphic areas (recurrent)	ND	ND	ND	ND	ND	*FUS*::*CREB3L2* (PCR)	Surgical resection	Recurrence, died of cardiac failure at 84 mo

M, male; F, female; LGFMS, low-grade fibromyxoid sarcoma; SEF, sclerosing epithelioid fibrosarcoma; “+”, positive; “−”, negative; IHC, immunohistochemistry; PCR, polymerase chain reaction; FISH, fluorescence *in situ* hybridization; NGS, next generation sequencing; NA, not available; ND, not done; NED, no evidence of disease; DOD, dead of disease; AWD, alive with disease; mo, month.

Histologically, classic LGFMS is composed of alternating fibrous and myxoid areas with the proliferation of bland, spindled cells in a whorling or short fascicular pattern. Almost 30% of LGFMS cases exhibit collagenous rosettes and a subset of cases harbor some unusual features, including the presence of sclerosing epithelioid fibrosarcoma (SEF)-like areas and epithelioid or round tumor cells with increased pleomorphism and atypia (3). In the reported 27 intrathoracic LGFMS cases, 26 cases had available histology information (including the current case). The morphologic results showed that 9 (9/26, 34.6%) cases were classic LGFMS and 13 (13/26, 50.0%) cases were LGFMS with a collagen rosette structure. In addition, 4 (4/26, 15.4%) cases had unusual features, consisting of 1 case with collagen rosettes and epithelioid component, 1 case with collagen rosettes and SEF-like areas, 1 lesion with round and epithelioid cell areas, and 1 case with classic morphology in the primary tumor and pleomorphic areas in the recurrent tumor (22, 29, 37, 42). The frequency of these unusual features in the intrathoracic LGFMS (15.4%) was slightly higher than that of the overall LGFMS cases (<10%) (1). Furthermore, the frequency of collagen rosettes in pulmonary LGFMS cases was particularly high, reaching 80% (8/9, 88.9%). These results revealed that giant collagen rosettes were more common in the intrathoracic cases, especially in the pulmonary lesions.

Immunohistochemically, MUC4 is a sensitive and specific marker for LGFMS, and 80% of LGFMS cases are positive for epithelial membrane antigen (EMA) and 30% show positivity for SMA (43). The current case was positive for MUC4 and negative for EMA and SMA. In the historical intrathoracic LGFMS cases, MUC4 immunohistochemistry was carried out in 4 cases and all of them displayed positivity, indicating that MUC4 immunostaining is a useful ancillary diagnostic tool (7, 23, 33, 40). Notably, MUC4 could also show positivity in SEF and fusion-positive alveolar rhabdomyosarcomas (44). In such difficult cases, molecular testing for *FUS* gene rearrangement could be helpful.

Cytogenetically, over 90% of LGFMSs harbor *FUS* gene rearrangement, with *FUS::CREB3L2* or *FUS::CREB3L1* fusion gene. Additionally, a subset of LGFMSs have *EWSR1* gene rearrangement, with *EWSR1::CREB3L1* fusion (5, 6). Our present case was the first pleural case with confirmed *FUS* gene rearrangement and *FUS::CREB3L2* fusion transcript. Eleven historical intrathoracic cases had molecular results, including 3 cases with *FUS* rearrangement and 5 cases with *FUS::CREB3L2* fusion gene. The genetic analysis of 2 cases was performed for differential diagnosis, and another 1 case was proved to be negative for *FUS* rearrangement but positive for MUC4 immunostaining (24, 28, 29, 31, 37, 40–42). MUC4 could also be positive in other lesions, such as in alveolar rhabdomyosarcomas (44). In such cases, FISH for *EWSR1* rearrangement and NGS would be helpful to confirm the diagnosis. Moreover, we endorsed genetic analysis for cases with ambiguous morphology in such rare locations.

It is worth noting that the current case exhibited extraordinarily rare clinicopathological features. First, the pleura is an extremely rare location for LGFMSs. Second, LGFMS is exceedingly rare in this age group (under 5 years old). Third, collagen rosettes were absent in the present case, which seemed to be common in the intrathoracic LGFMS cases. Hence, the differential diagnosis of this case is extremely challenging, and this tumor must be distinguished from other fibrous or myxoid lesions.

Inflammatory myofibroblastic tumor (IMT) often occurs in the pleuropulmonary area, outnumbering LGFMS. The current tumor was diffusely positive for MUC4 and negative for ALK, which is usually positive in IMT tumors. More importantly, IMT can be excluded because of the presence of the *FUS::CREB3L2* fusion gene in the current case, as IMT cases mainly harbor the rearrangement of the *ALK* gene (45).

Solitary fibrous tumor (SFT) is another common soft tissue tumor in this area and shares some morphologic features with LGFMS. However, the identification of MUC4 positivity could exclude the diagnosis of SFT. Furthermore, the genetic hallmark of SFT is the *NAB2::STAT6*, and LGFMS is featured by the *FUS* gene rearrangement (46).

Synovial sarcoma (SS) can also arise in the pleuropulmonary sites. Nevertheless, SSs harbor more conspicuous cellularity and positivity with EMA, TLE1, and cytokeratins. Cytogenetically, SSs are characterized by the t(X;18) (p11.2;q11.2) translocation, which leads to *SS18-SSX* gene fusion (47).

Desmoid fibromatosis with myxoid change could be also confused with LGFMS. However, almost 80% of desmoid fibromatosis was positive for β-catenin, which was negative in the present case (48). The majority of desmoid fibromatosis harbors somatic *CTNNB1* gene mutation, and a subset of cases arising in Gardner syndrome patients have *APC* gene mutation, which was different from the genetic hallmark of LGFMS.

Although dedifferentiated liposarcoma (DDL) rarely occurs in the thorax, it is necessary to differentiate LGFMS from DDL, as the two entities have different behaviors. DDL is usually positive for MDM2 and CDK4 immunostaining and characterized by the amplification of the *MDM2* gene, while the current case was negative for *MDM2* amplification (49).

The histology of malignant peripheral nerve sheath tumor (MPNST) and LGFMS can overlap significantly. However, our case was positive for MUC4 immunostaining but negative for S100 protein and without loss of H3K27me3, which are useful diagnostic biomarkers for MPNST (50). The identification of *FUS* rearrangement further demonstrated the diagnosis as LGFMS.

The current case also needed to be discriminated from SEF. However, classic SEF was composed of bland, monomorphic epithelioid cells, arranged in cords or nests within a densely collagenous stroma, while such morphologic changes were not identified in our case. Furthermore, the majority of SEFs harbor the *EWSR1::CREB3L1* fusion gene, while the present case exhibited *FUS::CREB3L2* fusion (51). Moreover, the recent study found that LGFMS and SEF could be classified by the different methylation profiles of the two entities (52).

Surgical resection is the main therapy for LGFMS, and our case showed no evidence of the disease after 11 months of the surgery. In 27 previously reported intrathoracic LGFMS cases, follow-up information was available in 16 cases who received surgical resection (including the current case), with a median follow-up of 23 months (range 6–110 months) (7, 22, 26, 30, 32–34, 38, 40–42). Three patients (3/16, 18.8%) developed recurrence, and none of the patients developed metastasis. The outcome results showed that 14 patients (87.5%) were alive without disease, 1 patient was alive with disease, and 1 patient died of cardiac failure. Long-term follow-up is still needed, as Evans et al. found that recurrence, metastasis rates, and mortality were 64%, 45%, and 42% of LGFMS with long-term follow-up (2). Additionally, LGFMSs with SEF-like areas and round-cell morphologic changes were reported to have more aggressive behavior (2). Four previous intrathoracic LGFMS cases had such morphologic change, and three of them had available follow-up information: one case was alive without disease, one case died of cardiac failure, and one case died of pericardial effusion and tamponade (22, 29, 37, 42). Hence, more cases are needed to verify the relation between the SEF-like and round-cell components with the behavior and prognosis of LGFMS cases.

In summary, we presented an extremely rare primary pleural LGFMS in a child with detailed clinicopathological and genetic results and carefully reviewed the literature on intrathoracic cases. To the best of our knowledge, the present case is the first genetically confirmed primary pleural LGFMS in a 4-year-old child with the identification of the *FUS::CREB3L2* fusion gene. Our study expands the age range of pleural LGFMS and highlights the use of immunohistochemical and molecular analyses in such challenging cases.

## Data availability statement

The original contributions presented in the study are included in the article/supplementary material. Further inquiries can be directed to the corresponding author.

## Ethics statement

Written informed consent was obtained from the minor(s)’ legal guardian/next of kin for the publication of any potentially identifiable images or data included in this article.

## Author contributions

XNH: Writing – original draft. WJ: Writing – original draft. XH: Writing – review & editing. MC: Writing – original draft. HZ: Writing – review & editing, Conceptualization, Funding acquisition, Project administration, Supervision.

## References

[1] Doyle LA Mertens F . Low-grade fibromyxoid sarcoma. In: WHO Classification of Tumours Editorial Board, editors. World Health Organization classification of soft tissue and bone tumours, 5th ed. Lyon: IARC Press (2020). p. 127–9.

[2] Evans HL . Low-grade fibromyxoid sarcoma: a clinicopathologic study of 33 cases with long-term follow-up. Am J Surg Pathol (2011) 35:1450–62. doi: 10.1097/PAS.0b013e31822b3687 21921785

[3] Folpe AL Lane KL Paull G Weiss SW . Low-grade fibromyxoid sarcoma and hyalinizing spindle cell tumor with giant rosettes: a clinicopathologic study of 73 cases supporting their identity and assessing the impact of high-grade areas. Am J Surg Pathol (2000) 24:1353–60. doi: 10.1097/00000478-200010000-00004 11023096

[4] Matsuyama A Hisaoka M Shimajiri S Hayashi T Imamura T Ishida T . Molecular detection of FUS-CREB3L2 fusion transcripts in low-grade fibromyxoid sarcoma using formalin-fixed, paraffin-embedded tissue specimens. Am J Surg Pathol (2006) 30:1077–84. doi: 10.1097/01.pas.0000209830.24230.1f 16931951

[5] Mertens F Fletcher CD Antonescu CR Coindre JM Colecchia M Domanski HA . Clinicopathologic and molecular genetic characterization of low-grade fibromyxoid sarcoma, and cloning of a novel FUS/CREB3L1 fusion gene. Lab Invest (2005) 85:408–15. doi: 10.1038/labinvest.3700230 15640831

[6] Lau PP Lui PC Lau GT Yau DT Cheung ET Chan JK . EWSR1-CREB3L1 gene fusion: a novel alternative molecular aberration of low-grade fibromyxoid sarcoma. Am J Surg Pathol (2013) 37:734–8. doi: 10.1097/PAS.0b013e31827560f8 23588368

[7] Perez D El-Zammar O Cobanov B Naous R . Low-grade fibromyxoid sarcoma: A rare case in an unusual location. SAGE Open Med Case Rep (2020) 8:2050313X20944315. doi: 10.1177/2050313X20944315 PMC7436804 32874586

[8] Liang W Xu S . Imaging findings from a case of pleural low-grade fibromyxoid sarcoma similar to mesothelioma with pleural effusion. Clin Respir J (2016) 10:120–4. doi: 10.1111/crj.12175 24991993

[9] Kim SY Kim MY Hwang YJ Han YH Seo JW Kim YH . Low-grade fibromyxoid sarcoma: CT, sonography, and MR findings in 3 cases. J Thorac Imaging (2005) 20:294–7. doi: 10.1097/01.rti.0000171420.81428.16 16282909

[10] Ronen S Ko JS Rubin BP Kilpatrick SE Wang WL Lazar AJ . Superficial low-grade fibromyxoid sarcoma. J Cutan Pathol (2023) 50:147–54. doi: 10.1111/cup.14325 PMC10091772 36074249

[11] Ud Din N Ahmad Z Zreik R Horvai A Folpe AL Fritchie K . Abdominopelvic and retroperitoneal low-grade fibromyxoid sarcoma: A clinicopathologic study of 13 cases. Am J Clin Pathol (2018) 149:128–34. doi: 10.1093/ajcp/aqx137 29385413

[12] White IK Scherer AG Baumanis MM Abdulkader M Fulkerson DH . Rapidly enlarging low-grade fibromyxoid sarcoma with intracranial extension in a 5-year-old girl: case report. J Neurosurg Pediatr (2015) 16:372–6. doi: 10.3171/2015.3.PEDS14564 26140292

[13] Kurisaki-Arakawa A Suehara Y Arakawa A Takagi T Takahashi M Mitani K . Deeply located low-grade fibromyxoid sarcoma with FUS-CREB3L2 gene fusion in a 5-year-old boy with review of literature. Diagn Pathol (2014) 9:163. doi: 10.1186/s13000-014-0163-2 25183312 PMC4167136

[14] Dobin SM Malone VS Lopez L Donner LR . Unusual histologic variant of a low-grade fibromyxoid sarcoma in a 3-year-old boy with complex chromosomal translocations involving 7q34, 10q11.2, and 16p11.2 and rearrangement of the FUS gene. Pediatr Dev Pathol (2013) 16:86–90. doi: 10.2350/12-07-1225-CR.1 23075075

[15] Menon S Krivanek M Cohen R . Low-grade fibromyxoid sarcoma, a deceptively benign tumor in a 5-year-old child. Pediatr Surg Int (2012) 28:211–3. doi: 10.1007/s00383-011-3024-z 22130782

[16] Wu X Petrovic V Torode IP Chow CW . Low grade fibromyxoid sarcoma: problems in the diagnosis and management of a Malignant tumour with bland histological appearance. Pathology (2009) 41:155–60. doi: 10.1080/00313020802579276 19152188

[17] Rando G Buonuomo V D'Urzo C Vecchio F Caldarelli M Pintus C . Fibromyxoid sarcoma in a 4-year-old boy: case report and review of the literature. Pediatr Surg Int (2005) 21:311–2. doi: 10.1007/s00383-005-1400-2 15747125

[18] Billings SD Giblen G Fanburg-Smith JC . Superficial low-grade fibromyxoid sarcoma (Evans tumor): a clinicopathologic analysis of 19 cases with a unique observation in the pediatric population. Am J Surg Pathol (2005) 29:204–10. doi: 10.1097/01.pas.0000146014.22624.8e 15644777

[19] Canpolat C Evans HL Corpron C Andrassy RJ Chan K Eifel P . Fibromyxoid sarcoma in a four-year-old child: case report and review of the literature. Med Pediatr Oncol (1996) 27:561–4. doi: 10.1002/(sici)1096-911x(199612)27:6<561::aid-mpo10>3.0.co;2-b 8888818

[20] Li MT Chen HJ Shi DC Chen M Zhang Z Zhang HY . Low-grade fibromyxoid sarcoma: a clinicopathologic and molecular study of 10 genetically confirmed cases. Int J Clin Exp Pathol (2018) 11:5860–8. PMC6963072 31949672

[21] Park YH Kim CH Kim JH Park JE Yim SY . Rare concurrence of congenital muscular torticollis and a Malignant tumor in the same sternocleidomastoid muscle. Ann Rehabil Med (2018) 42:189–94. doi: 10.5535/arm.2018.42.1.189 PMC5852225 29560341

[22] Ershadi R Vahedi M Jahanbin B Tabatabaei FS Rafieian S . Giant primary low-grade fibromyxoid sarcoma arising from the left pulmonary parenchyma: A case report and literature review. Cancer Rep (Hoboken) (2022) 5:e1718. doi: 10.1002/cnr2.1718 36148539 PMC9675389

[23] Yoshimura R Nishiya M Yanagawa N Deguchi H Tomoyasu M Kudo S . Low-grade fibromyxoid sarcoma arising from the lung: A case report. Thorac Cancer (2021) 12:2517–20. doi: 10.1111/1759-7714.14107 PMC8447909 34374195

[24] Bartuma H Moller E Collin A Domanski HA Von Steyern FV Mandahl N . Fusion of the FUS and CREB3L2 genes in a supernumerary ring chromosome in low-grade fibromyxoid sarcoma. Cancer Genet Cytogenet (2010) 199:143–6. doi: 10.1016/j.cancergencyto.2010.02.011 20471519

[25] Sargar K Kao SC Spunt SL Hawkins DS Parham DM Coffin C . MRI and CT of low-grade fibromyxoid sarcoma in children: A report from children's oncology group study ARST0332. AJR Am J Roentgenol (2015) 205:414–20. doi: 10.2214/AJR.14.13972 PMC4570741 26204295

[26] Oramas DM Alqaidy D Moran CA . Primary pulmonary hyalinizing spindle cell tumor with giant rosettes: A clinicopathological and immunohistochemical study of 2 cases. Ann Diagn Pathol (2021) 51:151706. doi: 10.1016/j.anndiagpath.2021.151706 33516059

[27] Magro G Fraggetta F Manusia M Mingrino A . Hyalinizing spindle cell tumor with giant rosettes: a previously undescribed lesion of the lung. Am J Surg Pathol (1998) 22:1431–3. doi: 10.1097/00000478-199811000-00018 9808138

[28] Kim L Yoon YH Choi SJ Han JY Park IS Kim JM . Hyalinizing spindle cell tumor with giant rosettes arising in the lung: report of a case with FUS-CREB3L2 fusion transcripts. Pathol Int (2007) 57:153–7. doi: 10.1111/j.1440-1827.2006.02073.x 17295648

[29] Whale K Bennett G . Primary pulmonary hyalinising spindle cell tumour with giant rosettes. Pathology (2014) 46:451–3. doi: 10.1097/pat.0000000000000128 24977731

[30] Kurul İ.C Celik A Akyurek N Teber I Demiroz M Memis L . A rare Malign tumor of the lung; low-grade fibromyxoid sarcoma: case report. J Clin Analytical Med (2012) 3:356–8. doi: 10.4328/JCAM.546

[31] Mustafa S VandenBussche CJ Ali SZ Siddiqui MT Wakely PE Jr . Cytomorphologic findings of low-grade fibromyxoid sarcoma. J Am Soc Cytopathol (2020) 9:191–201. doi: 10.1016/j.jasc.2020.01.006 32197967

[32] Xie Y Wang S Yan D Shen J . Primary low-grade fibromyxoid sarcoma of the mediastinum: A case report. Asian J Surg (2022) 45:2150–1. doi: 10.1016/j.asjsur.2022.05.005 35584992

[33] Sajid MI Arshad S Abdul-Ghafar J Fatimi SH Din NU . Low-grade fibromyxoid sarcoma incidentally discovered as an asymptomatic mediastinal mass: a case report and review of the literature. J Med Case Rep (2021) 15:50. doi: 10.1186/s13256-020-02605-4 33526082 PMC7851906

[34] Maeda E Ohta S Watadani T Goto A Nakajima A Ohtomo K . Imaging findings of thoracic low-grade fibromyxoid sarcoma: report of three cases. Jpn J Radiol (2009) 27:375–80. doi: 10.1007/s11604-009-0351-2 19943150

[35] Takanami I Takeuchi K Naruke M . Low-grade fibromyxoid sarcoma arising in the mediastinum. J Thorac Cardiovasc Surg (1999) 118:970–1. doi: 10.1016/s0022-5223(99)70076-0 10534712

[36] Aissaoui R Nasri S Abdelouahab H Mahjouba H Aichouni N Afkir S . Low-grade fibromyxoid sarcoma arising in the mediastinum: Case report and review of the literature. Radiol Case Rep (2022) 17:4814–7. doi: 10.1016/j.radcr.2022.09.030 PMC9550846 36238210

[37] Williams CM Du W Mangano WE Mei L . Mediastinal low-grade fibromyxoid sarcoma with FUS-CREB3L2 gene fusion. Cureus (2021) 13:e15606. doi: 10.7759/cureus.15606 34277226 PMC8273027

[38] Galetta D Cesario A Margaritora S Granone P . Primary mediastinal hyalinizing spindle cell tumor with giant rosettes. Ann Thorac Surg (2004) 77:2206–9. doi: 10.1016/S0003-4975(03)01388-2 15172307

[39] Gülhan SŞE Karadayı Ş Aydın M Karaoğlanoğlu N . Low-grade fibromyxoid sarcoma in the mediastinum: a case report. Turkish J Thorac Cardiovasc Surg (2012) 20:374–6. doi: 10.5606/tgkdc.dergisi.2012.073

[40] Chan YC Kan ANC Yuen LYP Wan IYP Fung KKF Cheung YF . Case report: primary thoracic low-grade fibromyxoid sarcoma in a young girl presenting with mediastinal mass syndrome. Front Pediatr (2022) 10:885068. doi: 10.3389/fped.2022.885068 35783305 PMC9247646

[41] Jakowski JD Wakely PE Jr . Primary intrathoracic low-grade fibromyxoid sarcoma. Hum Pathol (2008) 39:623–8. doi: 10.1016/j.humpath.2007.08.017 18275982

[42] Ferlosio A Doldo E Polisca P Orlandi A . Low-grade fibromyxoid sarcoma: an unusual cardiac location. Cardiovasc Pathol (2013) 22:e15–e7. doi: 10.1016/j.carpath.2012.11.004 23290535

[43] Doyle LA Möller E Dal Cin P Fletcher CD Mertens F Hornick JL . MUC4 is a highly sensitive and specific marker for low-grade fibromyxoid sarcoma. Am J Surg Pathol (2011) 35:733–41. doi: 10.1097/PAS.0b013e318210c268 21415703

[44] Forgo E Hornick JL Charville GW . MUC4 is expressed in alveolar rhabdomyosarcoma. Histopathology (2021) 78:905–8. doi: 10.1111/his.14321 PMC8058254 33368602

[45] Antonescu CR Suurmeijer AJ Zhang L Sung YS Jungbluth AA Travis WD . Molecular characterization of inflammatory myofibroblastic tumors with frequent ALK and ROS1 gene fusions and rare novel RET rearrangement. Am J Surg Pathol (2015) 39:957–67. doi: 10.1097/PAS.0000000000000404 PMC4465992 25723109

[46] Papp S Dickson BC Chetty R . Low-grade fibromyxoid sarcoma mimicking solitary fibrous tumor: a report of two cases. Virchows Arch (2015) 466:223–8. doi: 10.1007/s00428-014-1684-5 25416841

[47] Lan T Chen H Xiong B Zhou T Peng R Chen M . Primary pleuropulmonary and mediastinal synovial sarcoma: a clinicopathologic and molecular study of 26 genetically confirmed cases in the largest institution of southwest China. Diagn Pathol (2016) 11:62. doi: 10.1186/s13000-016-0513-3 27401493 PMC4939734

[48] Bhattacharya B Dilworth HP Iacobuzio-Donahue C Ricci F Weber K Furlong MA . Nuclear beta-catenin expression distinguishes deep fibromatosis from other benign and Malignant fibroblastic and myofibroblastic lesions. Am J Surg Pathol (2005) 29:653–9. doi: 10.1097/01.pas.0000157938.95785.da 15832090

[49] Henricks WH Chu YC Goldblum JR Weiss SW . Dedifferentiated liposarcoma: a clinicopathological analysis of 155 cases with a proposal for an expanded definition of dedifferentiation. Am J Surg Pathol (1997) 21:271–81. doi: 10.1097/00000478-199703000-00002 9060596

[50] Qiu Y Jing W Zhou Y Chen H Chen M Zhang H . Unusual split green-orange signals in USP6 fluorescence *in situ* hybridization in a Malignant peripheral nerve sheath tumor with a novel NF1-SCIMP fusion: a potential diagnostic pitfall. Virchows Arch (2022) 480:1255–60. doi: 10.1007/s00428-021-03179-2 34409490

[51] Guillou L Benhattar J Gengler C Gallagher G Ranchere-Vince D Collin F . Translocation-positive low-grade fibromyxoid sarcoma: clinicopathologic and molecular analysis of a series expanding the morphologic spectrum and suggesting potential relationship to sclerosing epithelioid fibrosarcoma: a study from the French Sarcoma Group. Am J Surg Pathol (2007) 31:1387–402. doi: 10.1097/PAS.0b013e3180321959 17721195

[52] Koelsche C Schrimpf D Stichel D Sill M Sahm F Reuss DE . Sarcoma classification by DNA methylation profiling. Nat Commun (2021) 12:498. doi: 10.1038/s41467-020-20603-4 33479225 PMC7819999

